# Outer membrane vesicles produced by *Burkholderia cepacia* cultured with subinhibitory concentrations of ceftazidime enhance pro-inflammatory responses

**DOI:** 10.1080/21505594.2020.1802193

**Published:** 2020-08-15

**Authors:** Se Yeon Kim, Mi Hyun Kim, Joo Hee Son, Seung Il Kim, Sung Ho Yun, Kyeongmin Kim, Shukho Kim, Minsang Shin, Je Chul Lee

**Affiliations:** a Department of Microbiology, School of Medicine, Kyungpook National University, Daegu, Republic of Korea; b Drug & Disease Target Team, Korea Basic Science Institute, Ochang, Republic of Korea; c Department of Bio-Analytical Science, University of Science and Technology (UST), Daejeon, Republic of Korea

**Keywords:** *Burkholderia cepacia*, antibiotics, outer membrane vesicle, cytotoxicity, inflammatory response

## Abstract

Burkholderia cepacia

is an opportunistic pathogen that infects patients with debilitating underlying diseases. This study investigated the production of outer membrane vesicles (OMVs) by *B. cepacia* cultured with sub-minimum inhibitory concentrations (MICs) of antibiotics and examined their pathogenic roles both *in vitro* and *in vivo. B. cepacia* ATCC 25416 produced more OMVs under antibiotic stress conditions than controls. OMVs isolated from *B. cepacia* cultured in Luria-Bertani (LB) broth (OMVs/LB) induced cytotoxicity and the expression of pro-inflammatory cytokine genes in A549 cells in a dose-dependent manner. Host cell cytotoxicity and pro-inflammatory responses were significantly higher in A549 cells treated with *B. cepacia* OMVs cultured with 1/4 MIC of ceftazidime (OMVs/CAZ) than in the cells treated with OMVs/LB, OMVs cultured with 1/4 MIC of trimethoprim/sulfamethoxazole (OMVs/SXT), or OMVs cultured with 1/4 MIC of meropenem. Intratracheal injection of *B. cepacia* OMVs also induced histopathology *in vivo* in mouse lungs. Expressions of *IL-1β* and *TNF-α* genes were significantly up-regulatedin the lungs of mice treated with OMVs/CAZ compared to mice administered other OMVs; the expression of the *GRO-α* gene, however, was significantly up-regulated in OMVs/SXT. In conclusion, OMVs produced by *B. cepacia* under different antibiotic stress conditions induce different host responses that may contribute to the pathogenesis of *B. cepacia*.

## Introduction

The *Burkholderia cepacia* complex (BCC) is a group of Gram-negative, aerobic, lactose non-fermenting, motile, catalase-positive bacteria comprised of more than 20 different species [1,2]. BCC bacteria were originally isolated from plants, but have since emerged as important opportunistic human pathogens, as well [3,4]. BCC poses few medical consequences to immunocompetent hosts, but patients with underlying diseases such as cystic fibrosis (CF), chronic granulomatous disease, hematological malignancies, or chronic renal failure are susceptible to infection by these organisms [5–9]. Recently, BCC bacteria have emerged in hospital-acquired infections of immunocompromised patients unrelated to CF or chronic granulomatous disease. *B. cenocepacia* and *B. multivorans* are commonly isolated from patients with CF, whereas *B. cepacia* is the most common species among non-CF, immunocompromised patients [10,11]. Although *B. cepacia* is not commonly found in CF patients, colonization with *B. cepacia* has been associated with high morbidity and mortality in these patients [12,13]. Clinical isolates of BCC are often resistant to many commonly used antimicrobial agents, including aminoglycosides, anti-pseudomonal penicillins, cephalosporins, polymyxins, quinolones, and tetracyclines, due to intrinsic and acquired resistance [14–18]. Treatment regimens for BCC infections include ceftazidime (CAZ), minocycline, meropenem (MEM), or trimethoprim/sulfamethoxazole (SXT) [14,16–19]. In addition to antimicrobial resistance, BCC bacteria produce several extracellular products, including hemolysins, proteases, lipases, siderophores, and lipopolysaccharides (LPS), which assist in the initial stages of colonization and contribute to the progression of BCC infection [20–23]. However, virulence determinant secretions from bacteria and their interactions with host cells have yet to be well-characterized.

All eukaryotic and prokaryotic cells produce extracellular vesicles. Gram-negative bacteria produce outer membrane vesicles (OMVs) that contain proteins, LPS, nucleic acids, and other bacterial molecules [24,25]. OMVs engage in host-pathogen interactions by enabling transport of toxins and virulence factors, protection of the secreted cargo, biofilm formation, and modulation of the host immune response [26–28]. The production and molecular composition of OMVs varies with bacterial growth stage or among bacterial strains within the same species. Moreover, bacteria cultured in stressful environments such as antibiotic supplementation demonstrate increased OMV production *in vitro* [29–31]. Bacterial exposure to antibiotics, which is likely to occur *in vivo*, may influence OMV biogenesis and thereby modulate the host’s immune response.

BCC infection stimulates the innate immune responses of the host [32,33]. BCC infection can also aggravate CF clinical outcomes with recurrent exacerbations and enhancespro-inflammatory responses in the hosts, provoking the production of interleukin (IL)-1β, tumor necrosis factor (TNF)-α, and IL-8 [34]. Moreover, the cell-free supernatants of *B. cenocepacia* trigger the production of IL-8 in lung epithelial cells [35]. Since OMVs are nanocomplexes that contain diverse pathogen-associated molecular patterns (PAMPs) in their membranes and lumens [27,36], OMVs produced by BCC during bacterial colonization or infection may activate innate immune responses in the hosts. The production of OMVs by *B. cepacia*, especially under antibiotic stress conditions, and their impact on host pathology, however, remain uncharacterized. The aim of this study was to investigate OMV production by *B. cepacia* ATCC 25416 under antibiotic stress conditions and their contribution to the induction of innate immune responsesin an *in vitro* cell culture model and in an *in vivo* mouse model.

## Materials and methods

### Bacterial strains

*B. cepacia* ATCC 25416 from the American Type Culture Collection (Manassas, VA, USA) and two clinical *B. cepacia* isolates, P1311 and P1383, obtained from the Kyungpook National University Hospital Culture Collection for Pathogens (KNUH-CCP) were used in this study. The species identification of two clinical isolates was performed by VITEK II system (bioMérieux, Marcyl’Etoile, France) and *recA* sequencing. Bacteria were cultured in Luria-Bertani (LB) broth or LB broth supplemented with 1/4 minimum inhibitory concentration (MIC) of CAZ, MEM, and SXT at 37°C.

### Cell culture

Human lung epithelial A549 cells were purchased from the Korean Cell Line Bank (Seoul, Korea). Cells were grown in RPMI 1640 medium (HyClone, Logan, UT, USA) supplemented with 10% fetal bovine serum (HyClone), 2 mM l-glutamine, and 1,000 U/mL penicillin G 37°C in a humidified atmosphere with 5% CO_2_. Confluent cells were seeded in 6- and 96-well plates for the cytokine gene expression and cell viability assays, respectively.

### Antimicrobial susceptibility testing

The MICs of CAZ, MEM, and SXT (trimethoprim/sulfamethoxazole, 1/19), commonly used antimicrobial agents for treatment of *B. cepacia* infection, were determined by broth microdilution method according to the Clinical Laboratory Standards Institute (CLSI) [37]. *Escherichia coli* ATCC 25922 and *Pseudomonas aeruginosa* ATCC 27853 were used as quality control strains.

### Isolation of OMVs

The OMVs of *B. cepacia* were prepared from bacterial culture supernatants as previously described [31,38]. Bacteria were cultured with 500 mL of LB broth with shaking at 37°C. *B. cepacia* were cultured in 500 mL of LB broth supplemented with CAZ (16 μg/mL), MEM (2 μg/mL), and SXT (10 μg/mL) to isolate OMVs from bacteria under antibiotic stress conditions. Bacteria were cultured to 1.5 of optical density at A600 (OD_600_)(Supplementary Fig. S1). Bacterial cells were harvested by centrifugation (8,000 *g* for 15 min at 4°C), and supernatants were filtered using a bottle-top filter with a 0.22 μm membrane. The filtered supernatants were concentrated using a QuixStand Benchtop System (GE Healthcare, Amersham, UK) with a 500 kDa hollow fiber membrane (GE Healthcare). OMV samples were collected by ultracentrifugation at 150,000 *g* at 4°C for 3 h and then washed in phosphate-buffered saline (PBS) followed by another ultracentrifugation cycle. The OMV fractions were then resuspended in PBS. The protein concentration of OMVs was determined using a modified BCA assay (Thermo Scientific, Waltham, MA, USA). The purified OMVs were streaked on blood agar plates to check for sterility and then stored at −80°C until use.

### Sodium dodecyl sulfate-polyacrylamide gel electrophoresis (SDS-PAGE)

Cultured bacterial cells were lysed by sonication (Branson Ultrasonics Corp., Danbury, CT, USA). Proteins in the culture supernatant were precipitated with 10% trichloroacetic acid. The bacterial lysate, culture supernatants, and purified OMVs corresponding to 10 μg of protein were resuspended in SDS-PAGE sample buffer (1 M TrisHCl [pH 6.8], 10% SDS, 1% bromophenol blue, glycerol, and β-mercaptoethanol) and boiled for 10 min. The proteins were separated on a 12% SDS-PAGE gel, and gels were stained with Coomassie brilliant blue R-250 (Bio-Rad, Hercules, CA, USA).

### Transmission electron microscopic (TEM) analysis

Purified *B. cepacia* OMV samples were applied to copper grids, stained with 2% uranyl acetate, and then washed with PBS. The OMVs were visualized using a transmission electron microscope (Hitachi H-7500, Hitachi, Japan) operating at 120 kV.

### Nanoparticle tracking analysis (NTA)

The size and number of *B. cepacia* OMVs were measured using a NanoSight NS500 instrument with a 488 nm laser module and sCMOS camera module (Malvern Instruments, Worcestershire,UK) [39]. The purified OMVs were diluted in MilliQ water to a concentration of approximately 8–9 × 10^8^ particles/mL for 50–100 particles per frame in the NTA measurement. OMV samples were loaded in the sample chamber and then videos were recorded for 30s three times. The captured data were analyzed using NTA 3.1 software build 3.1.46. All measurements were performedin triplicate at room temperature.

### Cell viability test

The viability of A549 cells was measured using the 3-[4,5-dimethylthiazol-2-yl]-2,5 diphenyltetrazolium bromide (MTT) assay(Abcam, Cambridge, UK). Human lung epithelial A549 cells were used to analyze interactions with *B. cepacia*OMVs, as epithelial cells represent the first line of defense against bacteria or bacterial products. Cells were seeded at a concentration of 2.0 × 10^4^/well in a 96-well microplate. After treatment with different concentrations of *B. cepacia* OMVs for 24 h, cell viability was measured 2 h after treatment with MTT reagent at 590 nm. Cell viability was calculated as follows: cell viability (%) = the absorbance of OMV-treated cells/the absorbance of the control cells × 100%.

### RNA isolation and quantitative real-time polymerase chain reaction (qPCR) of pro-inflammatory cytokine and chemokine genes

The expression levels of genes encoding glyceraldehyde 3-phosphate dehydrogenase (GAPDH), IL-1β, IL-6, TNF-α, monocyte chemoattractant protein (MCP)-1, and IL-8 in human cells and growth regulated protein (GRO)-α in mice were determined by qPCR as described previously [40]. The primers for *GRO-α* gene were 5ʹ-TGT GGG AGG CTG TGT TTG TA-3ʹ and 5ʹ-ACG AGA CCA GGA GAA ACA GG-3ʹ. A549 cells were treated with various concentrations (5, 10, and 20 μg/mL) of *B. cepacia* OMVs for 6 h. Mice were sacrificed 24 h after OMV injection, and their lungs were removed. The right lung was used for extraction of RNA, with total RNA was extracted using an RNeasy Mini Kit (Qiagen, Valencia, CA, USA) according to the manufacturer’s instructions. Next, cDNA was synthesized by the reverse transcription of 2 μg of total RNA using oligo dT primers and TOPscript^TM^ reverse transcriptase (Enzynomics, Daejeon, Korea) in a total reaction volume of 20 μL. Gene transcripts were quantified using Power SYBR Green PCR Master Mix (Applied Biosystems, Foster City, CA, USA) in a StepOnePlus™ Real-Time PCR System (Applied Biosystems). The amplification specificity was evaluated by a melting curve analysis. Fold changes in gene expression were calculated using the comparative Ct method, and sample transcript levels were normalized to GAPDH expression levels. Each experiment was performed in triplicate.

### Intratracheal injection of OMVs in mice

Eight-week-old female BALB/c mice were maintained under specific-pathogen-free conditions. Neutropenia was induced via intraperitoneal injections of cyclophosphamide (150 mg/Kg) on days −4 and −1 before OMV injection [40,41]. Mice were anesthetized with avertin (Sigma Aldrich,St. Louis MO, USA) and five mice per group received intratracheal injections of *B. cepacia* OMVs (20 μg of OMVs dissolved in 100 μL of PBS). The control group was injected with the same volume of PBS alone. Mice were sacrificed 24 h after OMV injection, and both lungs were removed. The right lung was used to extract RNA; the left lung was stained with hematoxilin and eosin (H&E) to evaluate inflammatory responses. Animal experiments were performed two times independently. Animal experimental procedures were approved by the Animal Care Committee of Kyungpook National University.

### Statistical Analysis

Data were analyzed using R 3.3.4 (https://www.r-project.org/). Expression of cytokine genes was analyzed using a one-way analysis of variance (ANOVA) with Dunnett’s*post-hoc* test. Differences of *P* < 0.05 were considered statistically significant.

## Results

### Production of OMVs from B. cepacia during in vitro culture

**Figure 1.**
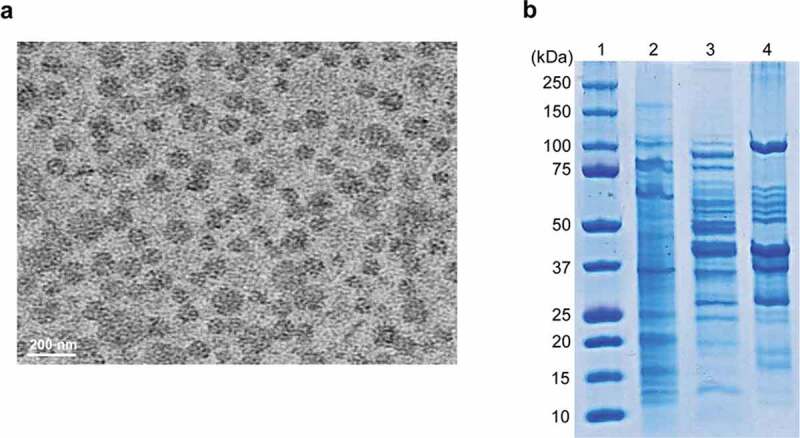
Production of OMVs in *B. cepacia* ATCC 25416. Bacteria were cultured in LB broth to late log phase, and OMVs were isolated from the culture supernatants. (a) Transmission electron micrographs. (b) SDS-PAGE analysis of bacterial proteins. Lane 1, molecular weight marker; 2, bacterial lysates; 3, culture supernatant; 4, OMVs.

To determine whether *B. cepacia* ATCC 25416 produced OMVs during *in vitro* culture, bacteria were cultured in LB broth to 1.5 at OD_600_, and OMVs were then isolated from the culture supernatant. TEM analysis showed that *B. cepacia* produced spherical OMVs (Figure 1(a)). SDS-PAGE analysis revealed different protein profiles among bacterial lysates, culture supernatants, and OMVs (Figure 1(b)). These results indicate that *B. cepacia* ATCC 25416 produces OMVs during *in vitro* culture.

### Responses to B. cepacia OMVs in A549 cells

**Figure 2.**
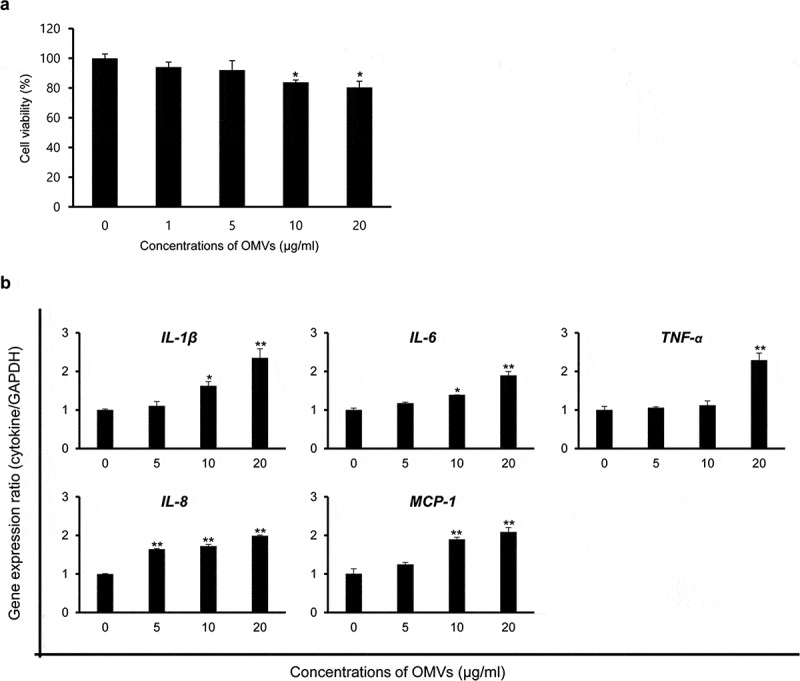
Host cell responses of *B. cepacia* OMVs in A549 cells. OMVs were isolated from *B. cepacia* ATCC 25416 cultured in LB broth to late log phase. (a) Cytotoxicity in A549 cells treated with *B. cepacia* OMVs. Cells were treated with various concentrations of *B. cepacia* OMVs for 24 h, and cell viability was determined by a MTT assay. Data are presented as mean ± SD of three independent experiments. *P < 0.05 compared to untreated control cells. (b) Expression of pro-inflammatory cytokine and chemokine genes in A549 cells treated with *B. cepacia* OMVs. Cells were treated with various concentrations of OMVs for 6 h and gene expression was assessed by qPCR. Data are presented as mean ± SD of three independent experiments. * *P* < 0.05, **P < 0.01 compared to untreated control cells.

To determine whether OMVs produced by *B. cepacia* ATCC 25416 cultured in LB broth (OMVs/LB) could induce cytotoxicity in host cells, A549 cells were treated with various concentrations (1 to 20 μg/mL protein concentrations) of OMVs/LB for 24 h, and cell viability was analyzed with a MTT assay. Cytotoxicity was slightly induced in A549 cells treated with ≥10 μg/mL of OMVs/LB (Figure 2(a)). Next, to determine whether *B. cepacia* OMVs could induce pro-inflammatory responses*in vitro*, A549 cells were treated with various concentrations (5, 10, and 20 μg/mL) of OMVs/LB for 6 h and qPCR was performed to analyze the expression of the pro-inflammatory cytokine genes *IL-1β, IL-6*, and *TNF-α*, and chemokine genes *IL-8* and *MCP-1*. No cytotoxicity was observed in A549 cells treated with ≤20 μg/mL of OMVs/LB for 6 h (data not shown). The expression of all tested cytokine genes was significantly increased in A549 cells treated with OMVs/LB, but the concentrations of OMVs/LB required to stimulate their expression varied among genes (Figure 2(b)). These results suggest that *B. cepacia* OMVs induce cytotoxicity and the expression of genes involved in pro-inflammatory responses in A549 cells.

### Production of OMVs by B. cepacia cultured with subinhibitory concentrations of antibiotics

**Figure 3.**
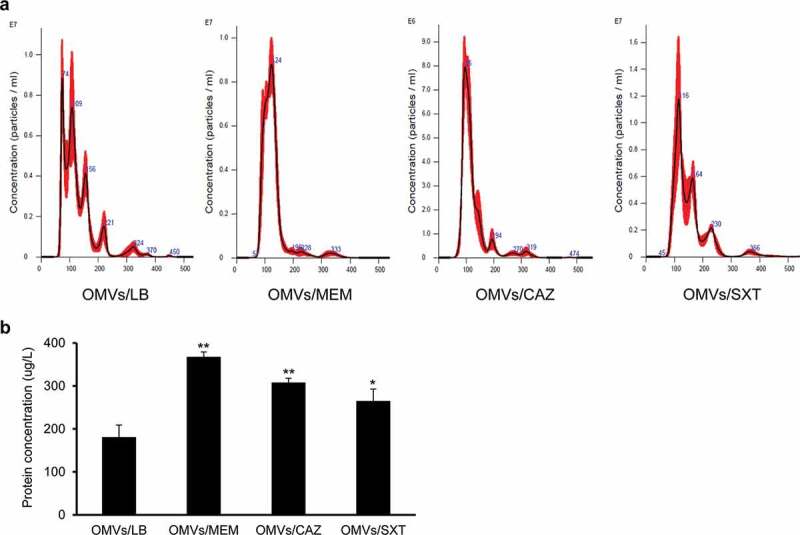
Production of OMVs from *B. cepacia* ATCC 25416 cultured with subinhibitory concentrations of antibiotics. OMVs were isolated from culture supernatants of *B. cepacia* cultured in LB (OMVs/LB), LB with 2 μg/mL meropenem (OMVs/MEM), LB with 16 μg/mLceftazidime (OMVs/CAZ), or LB with 0.5/9.5 μg/mL trimethoprim-sulfamethoxazole (OMVs/SXT). (a) The size and number of OMV particles were determined using nanoparticle tracking analysis. The data are representative of three independent experiments with similar results. (b) The protein concentration of OMVs isolated from 1 L of bacterial culture was measured using a modified BCA assay. The data are presented as mean ± SD of three independent experiments. * *P* < 0.05, **P < 0.01 compared to OMVs/LB.

To determine the effect of subinhibitory concentrations of antibiotics on OMV production in *B. cepacia, B. cepacia* ATCC 25416 was cultured in LB broth with 1/4 MIC of SXT (0.5/9.5 μg/mL), MEM (2 μg/mL), or CAZ (16 μg/mL), to 1.5 at OD_600_ (Supplementary Figure S1), and OMVs were then isolated from the culture supernatants. The sizes of OMVs/LB, OMVs from *B. cepacia* cultured with 1/4 MIC of MEM (OMVs/MEM), OMVs from *B. cepacia* cultured with 1/4 MIC of CAZ (OMVs/CAZ), and OMVs from *B. cepacia* cultured with 1/4 MIC of SXT (OMVs/SXT) were 129.7 ± 0.8 nm, 127.6 ± 1.2 nm, 123.4 ± 2.5 nm, and 154.9 ± 7.2 nm, respectively (Figure 3(a)). OMV samples obtained from 1 L culture of *B. cepacia* with LB, 1/4 MIC of MEM, 1/4 MIC of CAZ, and 1/4 MIC of SXT contained 2.79 × 10^12^, 2.45 × 10^13^, 1.91 × 10^13^, and 3.58 × 10^12^ particles, respectively. *B. cepacia* cultured with 1/4 MIC of MEM, CAZ, and SXT produced 2.0 (368 ± 11 μg/L), 1.7 (308 ± 10 μg/L), and 1.5 (265 ± 50 μg/L) times more OMV proteins than bacteria cultured without antibiotics (181 ± 28 μg/L), respectively (Figure 3(b)). These results suggest that subinhibitory concentrations of antibiotics increase the production of OMVs in *B. cepacia* ATCC 25416.

### Host cell cytotoxicity against OMVs from B. cepacia cultured with subinhibitory concentrations of antibiotics

**Figure 4.**
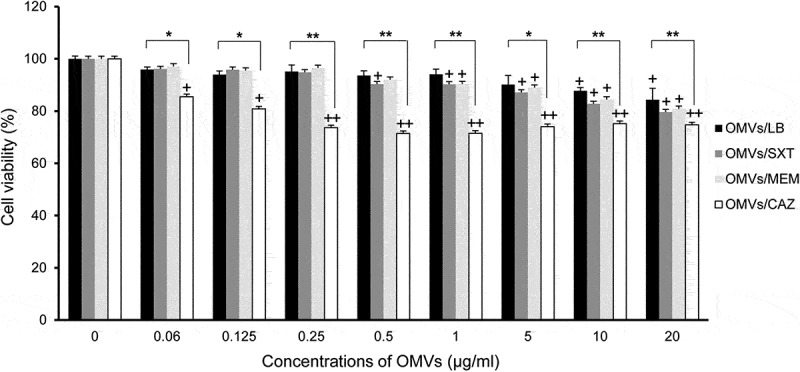
Cytotoxicity of A549 cells treated with OMVs from *B. cepacia* cultured with or without antibiotics. OMVs were isolated from culture supernatants of *B. cepacia* ATCC 25416 cultured in LB (OMVs/LB), LB with 0.5/9.5 μg/mL trimethoprim-sulfamethoxazole (OMVs/SXT), LB with 2 μg/mL meropenem (OMVs/MEM), or LB with 16 μg/mLceftazidime (OMVs/CAZ). Cells were treated with various concentrations of *B. cepacia* OMVs for 24 h, and cell viability was determined using the MTT assay. Data are presented as mean ± SD of three independent experiments. +*P* < 0.05, ++*P* < 0.01 compared to untreated control cells.*P < 0.05, **P < 0.01 compared to OMVs/LB.

The cytotoxic activity of OMVs/SXT, OMVs/MEM, and OMVs/CAZ was evaluated in A549 cells. Cytotoxicity was observed in A549 cells treated with ≥0.06 μg/mL of OMVs/CAZ, ≥0.5 μg/mL of OMVs/SXT, and ≥1.0 μg/mL of OMVs/MEM (Figure 4). Host cell cytotoxicity was significantly different between OMVs/LB and OMVs/CAZ at ≥0.06 μg/mL of protein and between OMVs/SXT or OMVs/MEM, and OMVs/CAZ at 0.25 to 5 μg/mL. These results suggest that OMVs/CAZ are more cytotoxic than other OMVs toward A549 cells.

### Expression of pro-inflammatory cytokine and chemokine genes in A549 cells treated with OMVs from B. cepacia cultured with subinhibitory concentrations of antibiotics

**Figure 5.**
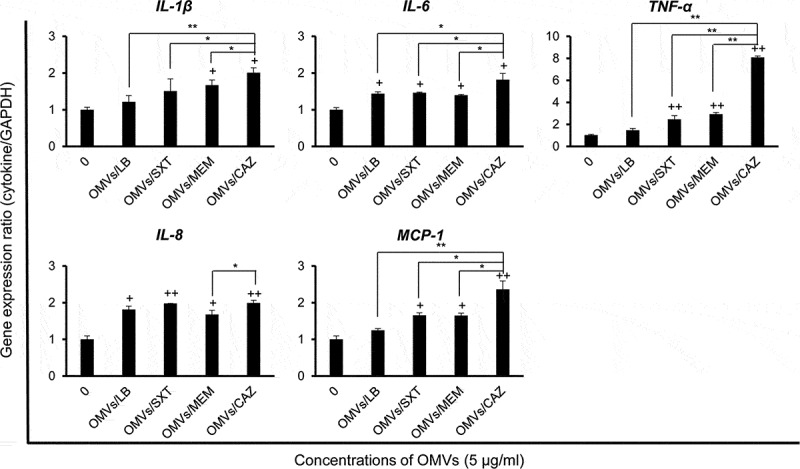
Expression of pro-inflammatory cytokine and chemokine genes in A549 cells treated with *B. cepacia* OMVs. OMVs were isolated from culture supernatants of *B. cepacia* ATCC 25416 cultured in LB (OMVs/LB), LB with 0.5/9.5 μg/mL trimethoprim-sulfamethoxazole (OMVs/SXT), LB with 2 μg/mL meropenem (OMVs/MEM), or LB with 16 μg/mL ceftazidime (OMVs/CAZ). Cells were treated with 5 μg/mL of OMVs for 6 h and gene expression was assessed by qPCR. Data are presented as mean ± SD of three independent experiments. +*P* < 0.05, ++*P* < 0.01 compared to untreated control cells.*P < 0.05, **P < 0.01 compared to OMVs/CAZ.

To investigate the effects of OMVs produced by *B. cepacia* ATCC 25416 cultured with subinhibitory concentrations of antibiotics on pro-inflammatory responses*in vitro*, A549 cells were treated with 5 μg/mL of OMVs/SXT, OMVs/MEM, or OMVs/CAZ for 6 h, and the expression of cytokine genes was measured using qPCR. OMVs/CAZ and OMVs/MEM stimulated the expression of all tested cytokine genes as compared to untreated control cells (Figure 5), but expression levels of all tested genes were not significantly different between OMVs/SXT and OMVs/MEM. The expression of *TNF-α* and *MCP-1* genes was significantly different between OMVs/LB and OMVs/SXT or OMVs/MEM. The expression levels of *IL-1β, IL-6, TNF-α*, and *MCP-1* genes were significantly different between OMVs/CAZ and other OMVs. Expression of the *IL-8* gene was significantly different between OMVs/MEM and OMVs/CAZ. These results suggest that OMVs/CAZ induce a strong pro-inflammatory response *in vitro*.

### Lung pathology induced by OMVs from B. cepacia cultured with subinhibitory concentrations of antibiotics

**Figure 6.**
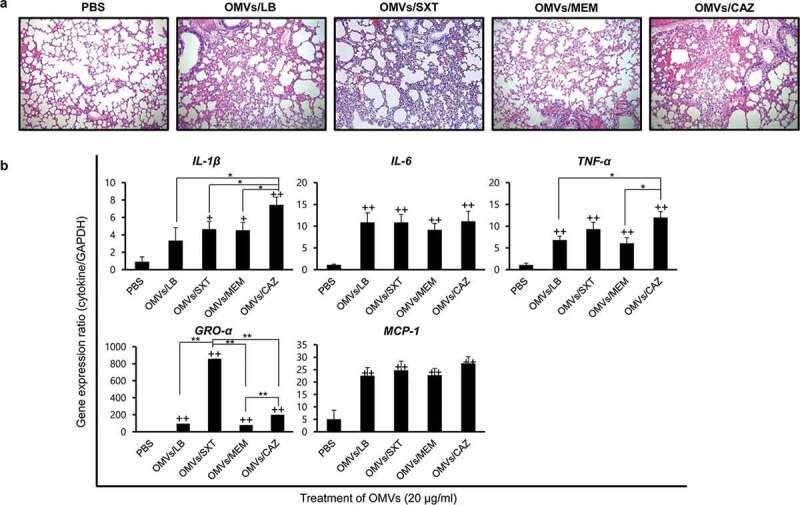
Inflammatory responsesin the lungs of mice administered *B. cepacia* OMVs. OMVs were isolated from culture supernatants of *B. cepacia* ATCC 25416 cultured in LB (OMVs/LB), LB with 0.5/9.5 μg/mL trimethoprim-sulfamethoxazole (OMVs/SXT), LB with 2 μg/mL meropenem (OMVs/MEM), or LB with 16 μg/mL ceftazidime (OMVs/CAZ). OMVs (20 μg of protein concentrations) were administered intratracheally, and mice were sacrificed 24 h after injection. (a) Histopathology of lungs. Lung tissues were stained by H&E. PBS was administered as a control. Magnification, 100X. (b) Pro-inflammatory response to *B. cepacia* OMVs in the lungs of mice. Lung tissues were removed, and gene expression was assessed by qPCR. Data are presented as the mean ± SD of five mice. +*P* < 0.05, ++*P* < 0.01 compared to PBS control group.*P < 0.05, **P < 0.01compared to OMVs/CAZ or OMVs/SXT.

To determine whether *B. cepacia* OMVs could elicit an inflammatory response *in vivo*, OMVs from *B. cepacia* ATCC 25416 were administered intratracheally in mice, and lungs were dissected 24 h after OMV injection. Neutropenic mice were used in this study, because *B. cepacia* commonly infected immunocompromised hosts and the patients with CF or chronic granulomatous diseases had defects in phagocyte function [10,11,42]. Mice were injected with PBS as a negative control. All mice injected with the four different *B. cepacia* OMVs, OMVs/LB, OMVs/SXT, OMVs/MEM, and OMVs/CAZ, survived for the full 24 h after treatment; however, inflammatory processes, including mild congestion and cellular infiltration, were observedin their lungs (Figure 6(a)). Four different *B. cepacia* OMVs elicited profound expression of pro-inflammatory cytokine *IL-1β, IL-6*, and *TNF-α* genes and chemokine *GRO-α* and *MCP-1* genes in the lungs compared to those from PBS control mice (Figure 6(b)). Expression of *IL-1β* and *TNF-α* genes was the most prominent in mice treated with OMVs/CAZ, whereas the expression of *GRO-α* gene was the most prominent in the mice treated with OMVs/SXT. These results suggest that *B. cepacia* OMVs can also induce the expression of genes involved in inflammatory responses *in vivo* and that OMVs from *B. cepacia* cultured with different antibiotics may induce different host inflammatory responses.

## Discussion

The production and secretion of OMVs from BCC bacteria during *in vitro* culture have been previously reported for clinical isolates of *B. cepacia* genomovar IIIa and *B. vietnamiensis* [43]. OMVs secreted from these microorganisms were between 30 and 220 nm in size by TEM analysis and contained several virulence factors, including lipases, phospholipases, proteases, peptidoglycan-degrading enzymes, and LPS. However, we did not find evidence that OMVs derived from BCC bacteria contributed to host pathology *in vitro* or *in vivo*. The present study showed that *B. cepacia* (genomovar I) secreted OMVs into the extracellular milieu during *in vitro* culture. *B. cepacia* cultured under antibiotic stress conditions such as SXT, MEM, or CAZ produced more OMVs than bacteria cultured without antibiotics. Moreover, OMVs produced by *B. cepacia* cultured with sub-MICs of antibiotics demonstrated greater cytotoxicity and more pronounced pro-inflammatory responses than OMVs produced by *B. cepacia* cultured without antibiotics. Of the tested antibiotics, CAZ culture resulted in the most profound cytotoxic and pro-inflammatory OMV effects both *in vitro* and *in vivo*. This study is the first to demonstrate that OMVs from *B. cepacia* cultured under different antibiotic stress conditions exhibit different ability to induce pro-inflammatory responses *in vitro* and *in vivo*.

We first examined the antimicrobial susceptibility and biofilm formation of three *B. cepacia* strains, ATCC 25416 isolated from onion and P1311 and P1383 isolated from human clinical specimens. *B. cepacia* ATCC 25416 showed higher biofilm-forming ability (Supplementary Figure S2) and higher MIC values of CAZ and SXT than two clinical *B. cepacia* isolates (Supplementary Table 1). Moreover, OMVs from *B. cepacia* ATCC 25416 showed more cytotoxic activity in A549 cells than OMVs from two clinical *B. cepacia* isolates (Supplementary Figure S3), although OMV production was not significantly different between *B. cepacia* strains. Cardona *et al* [44]. demonstrated that *B. cepacia* strains isolated from onion, including ATCC 25416, were more virulent than clinical *B. cepacia* strains from CF or chronic granulomatous disease patients using the mortality assays of *Caenorhabditis elegans*. Based on these results, we further evaluated the OMV production by *B. cepacia* ATCC 25416 under antibiotic stress conditions and their pathologic roles both *in vitro* and *in vivo*.

*B. cepacia* ATCC 25416 OMVs (≥10 μg/mL) obtained from control conditions (no antibiotics) did exert cytotoxic effects on A549 cells (Figures 2(a) and 4). OMVs derived from Gram-negative, non-fermenting bacteria, including *P. aeruginosa, Acinetobacter baumannii, Acinetobacter nosocomialis*, and *Stenotrophomonas maltophilia*, were also shown to induce cytotoxicity in epithelial cells *in vitro* [38,45–47]. OMVs (5 μg/mL) derived from *B. pseudomallei* did not induce cytotoxicity in murine macrophages [48]. Although host cell death induced by OMVs derived from different Gram-negative, non-fermenting bacterial species was highly dependent on cell type, cytotoxic concentrations of OMVs were all found to be 15 or 20 μg/mL protein concentrations [38,46,47]. Thus, cytotoxic activity of *B. cepacia* OMVs/LB did not vary significantly from that of OMVs from other Gram-negative non-fermenting bacteria. OMVs isolated from *B. cepacia* cultured under antibiotic stress conditions, however, did display greater cytotoxic effects on A549 cells than OMVs isolated from *B. cepacia* cultured without antibiotics (Figure 4). *B. cepacia* cultured with subinhibitory concentrations of CAZ produced 6.8 times more OMV particles than control bacteria, and these OMVs/CAZ were the most cytotoxic to A549 cells. These results suggest that subinhibitory concentrations of CAZ modulate the biogenesis of OMVs and stimulate the association of cytotoxic factors with *B. cepacia* OMVs. Azurin homologue in the culture supernatant of *B. cepacia* and hemolysin from *B. cepacia* genomovar III were found to be cytotoxic to host cells [49,50]. However, the present study did not determine the cytotoxic factors of *B. cepacia* OMVs and it is necessary to clarify cytotoxic factors packaged in *B. cepacia* OMVs and their association with host cell death.

OMVs carry diverse PAMPs derived from cell wall and cytoplasm, and these immunoreactive molecules can interact with host cells and stimulate inflammatory responses therein [26,27]. The present study showed that *B. cepacia* OMVs stimulated the expression of pro-inflammatory cytokine *IL-1β, IL-6*, and*TNF-α* genes and chemokine *IL-8* and *MCP-1* genes in A549 cells. In addition, pro-inflammatory responses and inflammation were observed in the lungs of mice treated with *B. cepacia* OMVs (Figure 6). OMVs derived from Gram-negative, non-fermenting, opportunistic pathogens, including *A. baumannii, A. nosocomialis, P. aeruginosa*, and *S. maltophilia*, stimulate the expression of pro-inflammatory cytokine genes in cultured epithelial cells [38,41,45–47]. Pulmonary inflammation was also observed in mice administered *A. baumannii* and *S. maltophilia* OMVs [41,47]. These data suggest that OMVs derived from Gram-negative, non-fermenting pathogens are a potent stimulator of inflammatory responsesboth *in vitro* and *in vivo*. In contrast, *B. pseudomallei* OMV immunization in mice reduces bacterial persistence and induces high titers of OMV-specific serum immunoglobulin (Ig) G and IgA [48]. Since inflammatory responses to bacteria or bacterial products and subsequent tissue damage are main pathogenic mechanisms of Gram-negative, non-fermenting, opportunistic pathogens, including *B. cepacia*, it is necessary to evaluate whether inflammatory responses against OMVs contribute to the eradication of invading bacteria, or instead enhance the pathogenicity of bacteria during infection.

The present study showed significant differences in the expression of pro-inflammatory cytokine and chemokine genes in A549 cells treated with OMVs isolated from *B. cepacia* cultured under differen tantibiotic stress conditions. OMVs/SXT and OMVs/MEM did not show significant difference in the expression of pro-inflammatory cytokine and chemokine genes in A549 cells, but the expression of *TNF-α* and *GRO-α* genes was significantly different between OMVs/SXT and OMVs/MEM in the lungs of experimental mice. Interestingly, the expression of all tested cytokine genes was at its highest in A549 cells treated with OMVs/CAZ (Figure 5). The expression of cytokine genes in A549 cells treated with OMVs/CAZ was highly correlated to that discovered in the lungs of mice injected with OMVs/CAZ, except for the *GRO-α* gene (Figure 6(b)). The expression of chemokine C-X-C motif ligand genesagainst OMVs/SXT was significantly different *in vitro* (*IL-8* in human cells) versus *in vivo* (*GRO-α* in mice). Greater expression of the *GRO-α* gene might be associated with the massive cellular infiltration visualized in the lung tissues of mice treated with OMVs/SXT (Figure 6(a)). These results suggest that OMVs produced by *B. cepacia* cultured with subinhibitory concentrations of CAZand SXT stimulate profound pro-inflammatory responses and recruitment of inflammatory cells, respectively, which may contribute to disease progression or clinical outcomes of patients infected with *B. cepacia*.

This study highlights the potential inflammatory consequences of OMVs produced by *B. cepacia*. Since antibiotics modulate bacterial physiology [51,52] and the biogenesis of OMVs at subinhibitory concentrations, the production of OMVs in *B. cepacia* under antibiotic stress conditions and their pathologic effects on host cells or tissues may improve our understanding of the functional importance of OMVs. Further studies are required to clarify the appropriateness of specific antibiotics in the treatment of patients infected with *B. cepacia*, as *B. cepacia* OMVs obtained from different antibiotic conditions have been shown to induce different inflammatory responses.

## Supplementary Material

Supplemental Material
