# Supplementary material for: Outer membrane vesicles produced by Burkholderia cepacia cultured with subinhibitory concentrations of ceftazidime enhance pro-inflammatory responses
Source: Virulence. 2020 Aug 15;11(1):995–1005. doi: 10.1080/21505594.2020.1802193 (PMC7567438; doi:10.1080/21505594.2020.1802193)
Supplement: Supplemental Material [file KVIR_A_1802193_SM4663.pptx]

## Slide 1
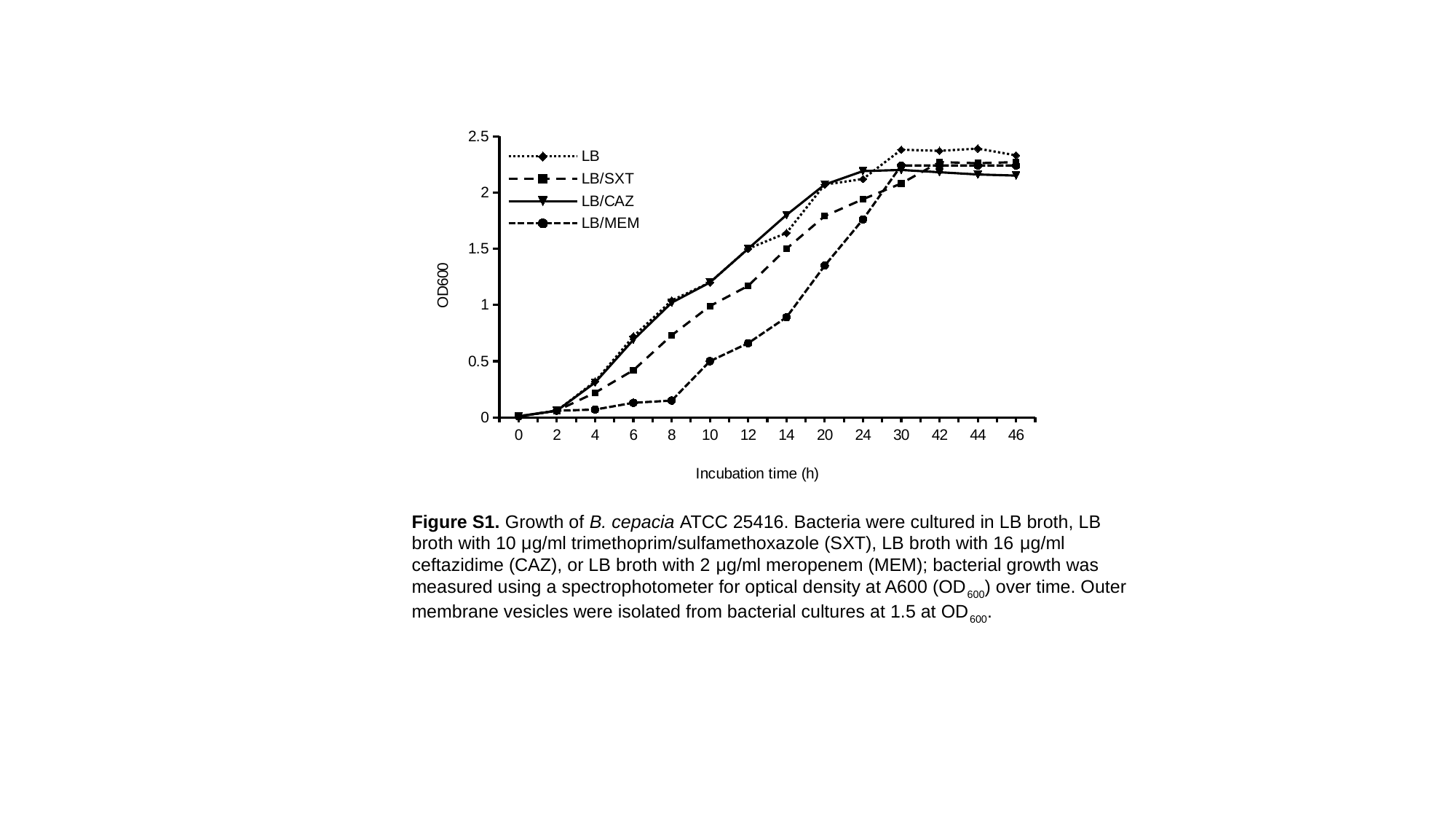

### Chart
| Category | LB | LB/SXT | LB/CAZ | LB/MEM |
|---|---|---|---|---|
| 0 | 0.010000000000000016 | 0.010000000000000016 | 0.010000000000000016 | 0.010000000000000016 |
| 2 | 0.06000000000000004 | 0.06000000000000004 | 0.06000000000000004 | 0.06000000000000004 |
| 4 | 0.3200000000000005 | 0.22000000000000006 | 0.31000000000000044 | 0.07000000000000003 |
| 6 | 0.7200000000000006 | 0.4200000000000003 | 0.6900000000000006 | 0.13 |
| 8 | 1.04 | 0.7300000000000006 | 1.02 | 0.15000000000000022 |
| 10 | 1.2 | 0.99 | 1.2 | 0.5 |
| 12 | 1.5 | 1.1700000000000017 | 1.5 | 0.6600000000000011 |
| 14 | 1.6400000000000001 | 1.5 | 1.8 | 0.8900000000000002 |
| 20 | 2.0700000000000003 | 1.7900000000000005 | 2.0700000000000003 | 1.35 |
| 24 | 2.12 | 1.9400000000000013 | 2.19 | 1.7600000000000005 |
| 30 | 2.38 | 2.08 | 2.2 | 2.24 |
| 42 | 2.3699999999999997 | 2.27 | 2.1799999999999997 | 2.24 |
| 44 | 2.3899999999999997 | 2.26 | 2.16 | 2.24 |
| 46 | 2.3299999999999987 | 2.27 | 2.15 | 2.24 |Figure S1. Growth of B. cepacia ATCC 25416. Bacteria were cultured in LB broth, LB broth with 10 μg/ml trimethoprim/sulfamethoxazole (SXT), LB broth with 16 μg/ml ceftazidime (CAZ), or LB broth with 2 μg/ml meropenem (MEM); bacterial growth was measured using a spectrophotometer for optical density at A600 (OD600) over time. Outer membrane vesicles were isolated from bacterial cultures at 1.5 at OD600.

## Slide 2
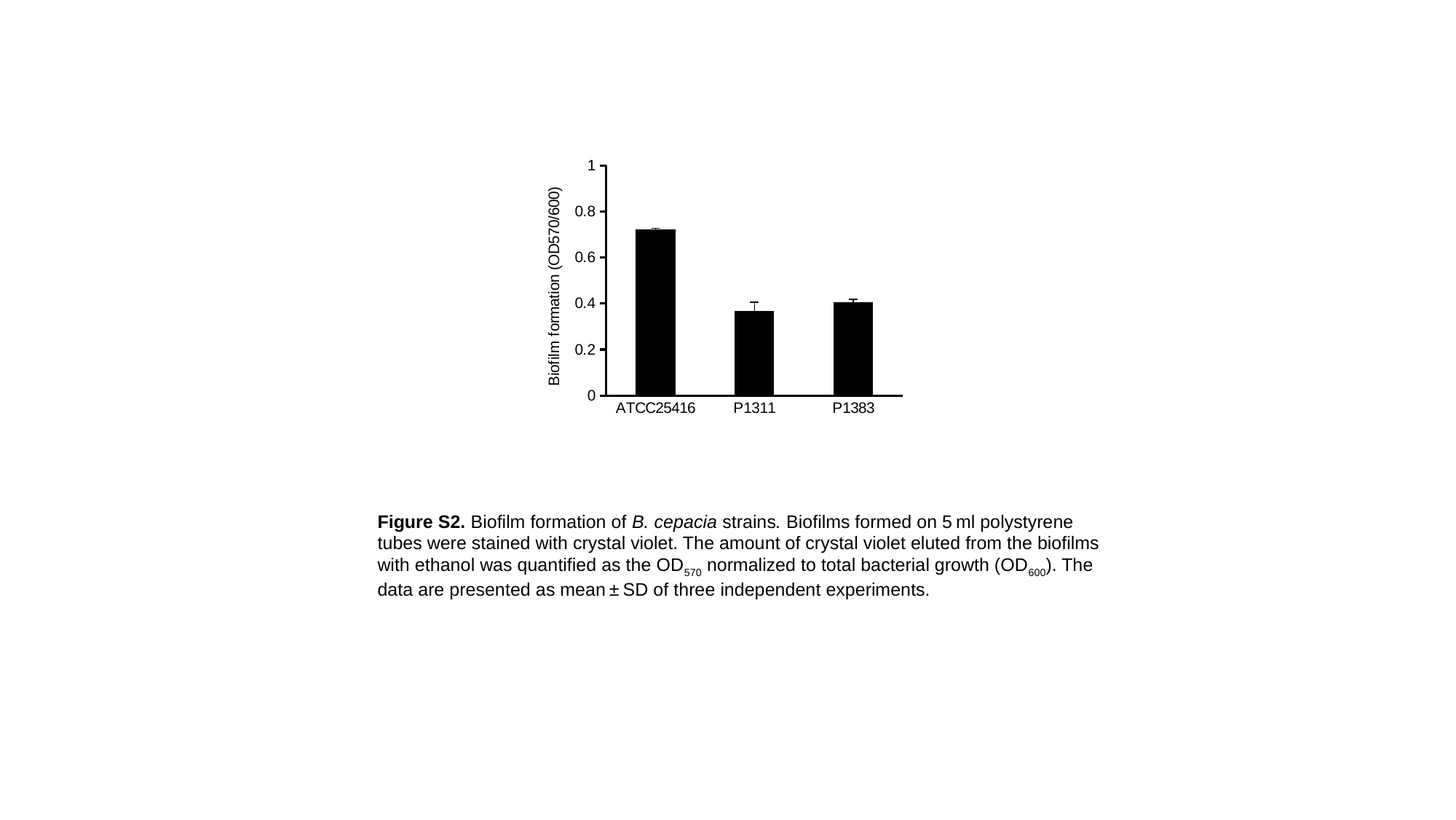

### Chart
| Category | |
|---|---|
| ATCC25416 | 0.7210942321103467 |
| P1311 | 0.3680730566170376 |
| P1383 | 0.40490642215092365 |Figure S2. Biofilm formation of B. cepacia strains. Biofilms formed on 5 ml polystyrene tubes were stained with crystal violet. The amount of crystal violet eluted from the biofilms with ethanol was quantified as the OD570 normalized to total bacterial growth (OD600). The data are presented as mean ± SD of three independent experiments.

## Slide 3
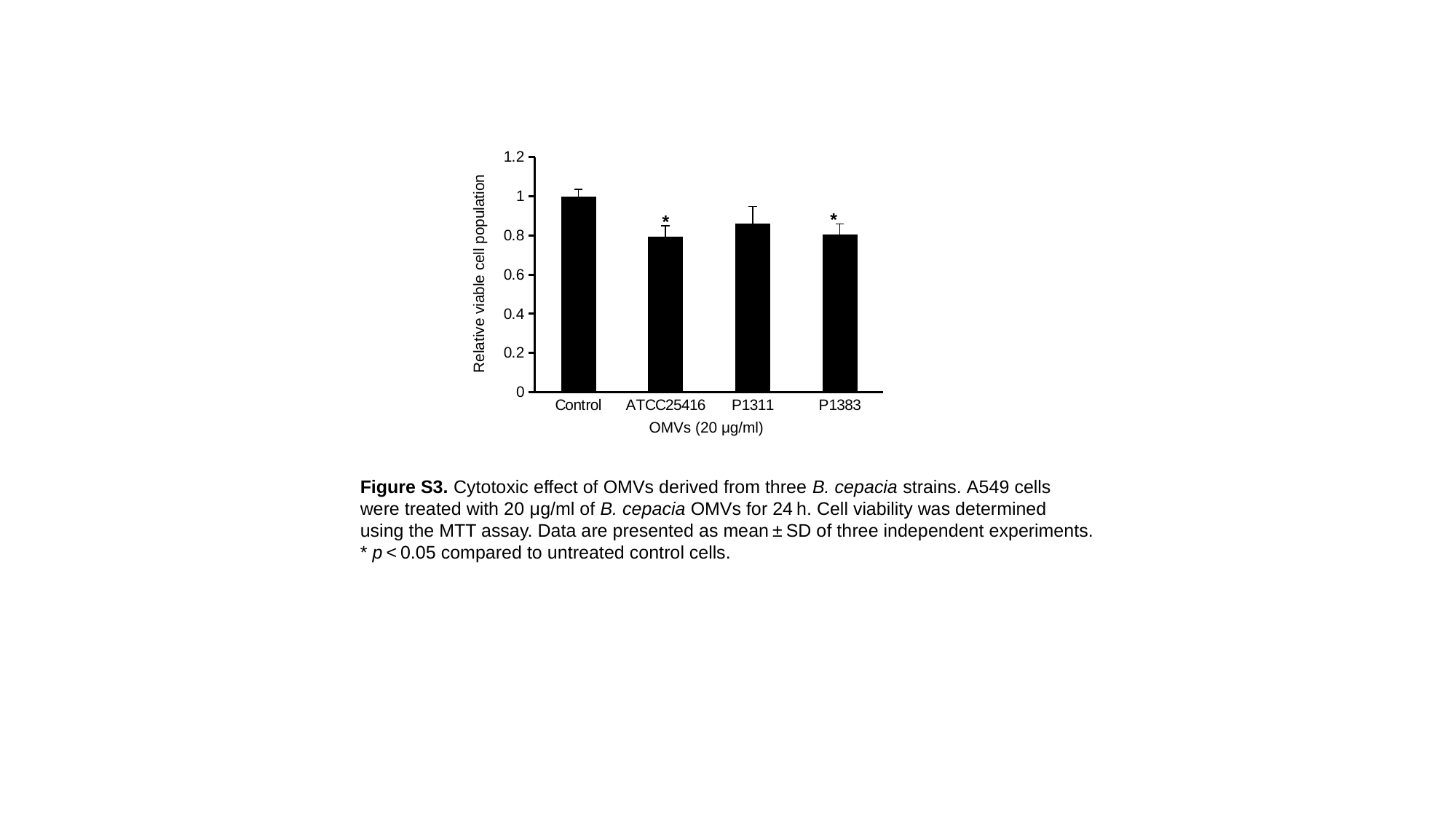

### Chart
| Category | |
|---|---|
| Control | 1.0000000000000002 |
| ATCC25416 | 0.7955043454711083 |
| P1311 | 0.8620940598562726 |
| P1383 | 0.8042010287060717 |*
*
Relative viable cell population
OMVs (20 μg/ml)
Figure S3. Cytotoxic effect of OMVs derived from three B. cepacia strains. A549 cells were treated with 20 μg/ml of B. cepacia OMVs for 24 h. Cell viability was determined using the MTT assay. Data are presented as mean ± SD of three independent experiments. * p < 0.05 compared to untreated control cells.

## Slide 4
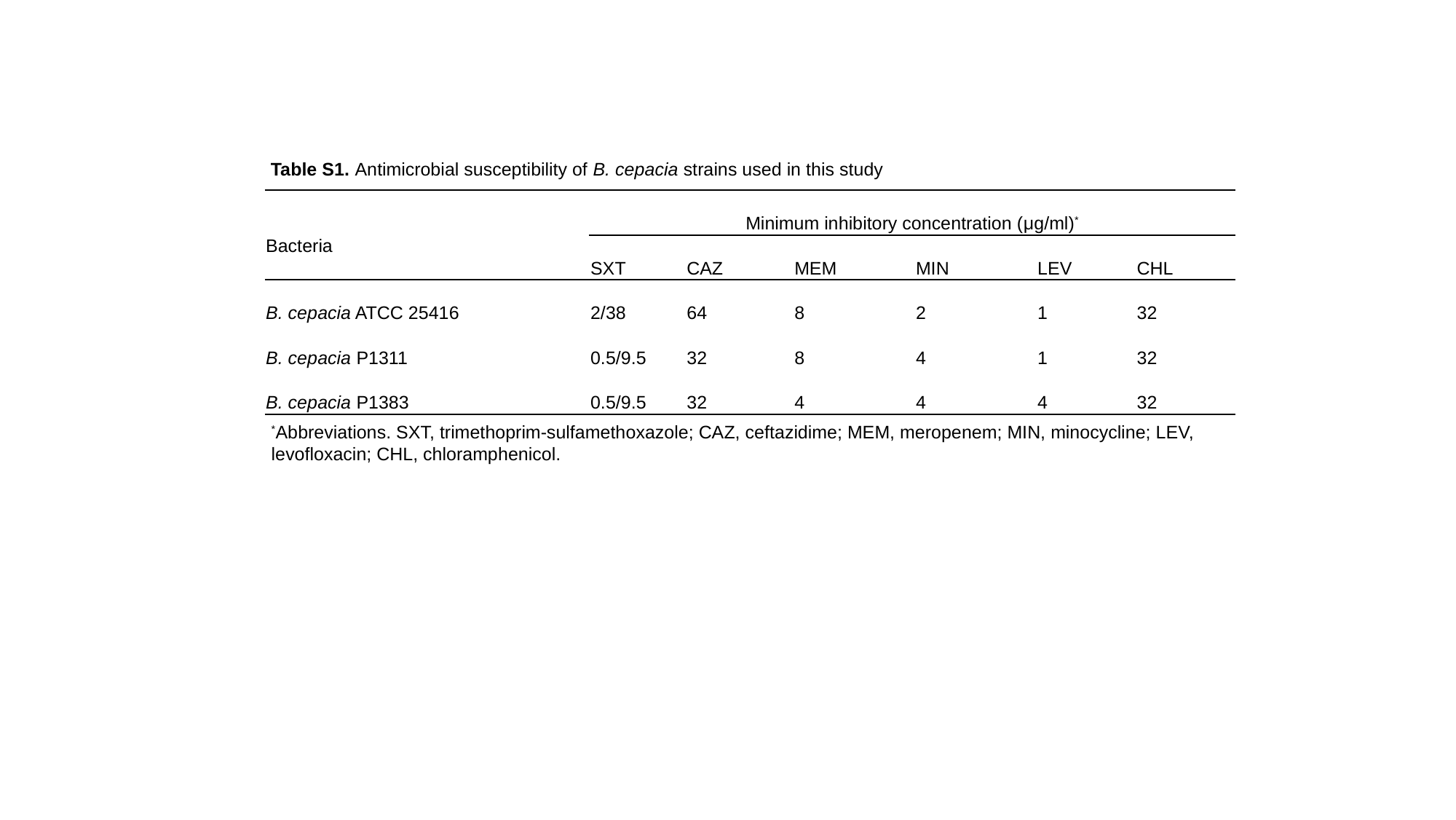

Table S1. Antimicrobial susceptibility of B. cepacia strains used in this study
| | | | | | | | |
| --- | --- | --- | --- | --- | --- | --- | --- |
| Bacteria | Minimum inhibitory concentration (μg/ml)\* | | | | | | |
| | SXT | | CAZ | MEM | MIN | LEV | CHL |
| B. cepacia ATCC 25416 | 2/38 | | 64 | 8 | 2 | 1 | 32 |
| B. cepacia P1311 | 0.5/9.5 | | 32 | 8 | 4 | 1 | 32 |
| B. cepacia P1383 | 0.5/9.5 | | 32 | 4 | 4 | 4 | 32 |
*Abbreviations. SXT, trimethoprim-sulfamethoxazole; CAZ, ceftazidime; MEM, meropenem; MIN, minocycline; LEV, levofloxacin; CHL, chloramphenicol.
